# Supplementary material for: Magnetic excitations beyond the single- and double-magnons
Source: Nat Commun. 2023 May 12;14:2749. doi: 10.1038/s41467-023-38341-8 (PMC10182046; doi:10.1038/s41467-023-38341-8)
Supplement: Supplementary file 1 — Supplementary Information [file 41467_2023_38341_MOESM1_ESM.pdf]

Supplementary Information for

Magnetic excitations beyond the single- and double-magnons

Hebatalla Elnaggar,[1,2]* Abhishek Nag,[3] Maurits W. Haverkort,[4] Mirian Garcia-Fernandez,[3] Andrew Walters,[3] Ru-Pan Wang,[1,5] Ke-Jin Zhou,[3]* Frank de Groot[1]*

[1]Debye Institute for Nanomaterials Science, Utrecht University, 3584 CA Utrecht, The Netherlands.
[2]Institute of Mineralogy, Physics of Materials and Cosmochemistry, CNRS, Sorbonne University, 4 Place Jussieu, 75005 Paris, France.
[3]Diamond Light Source, Harwell Campus, Didcot OX11 0DE, United Kingdom.
[4]Heidelberg University, Philosophenweg 19, 69120 Heidelberg, Germany.
[5]Department of Physics, University of Hamburg, Luruper Chaussee 149, G610, 22761 Hamburg,

*Corresponding author. Email: hebatalla.elnaggar@sorbonne-universite.fr, kejin.zhou@diamond.ac.uk, F.M.F.deGroot@uu.nl

This PDF file includes:

Figs. S1 to S4
Table S1

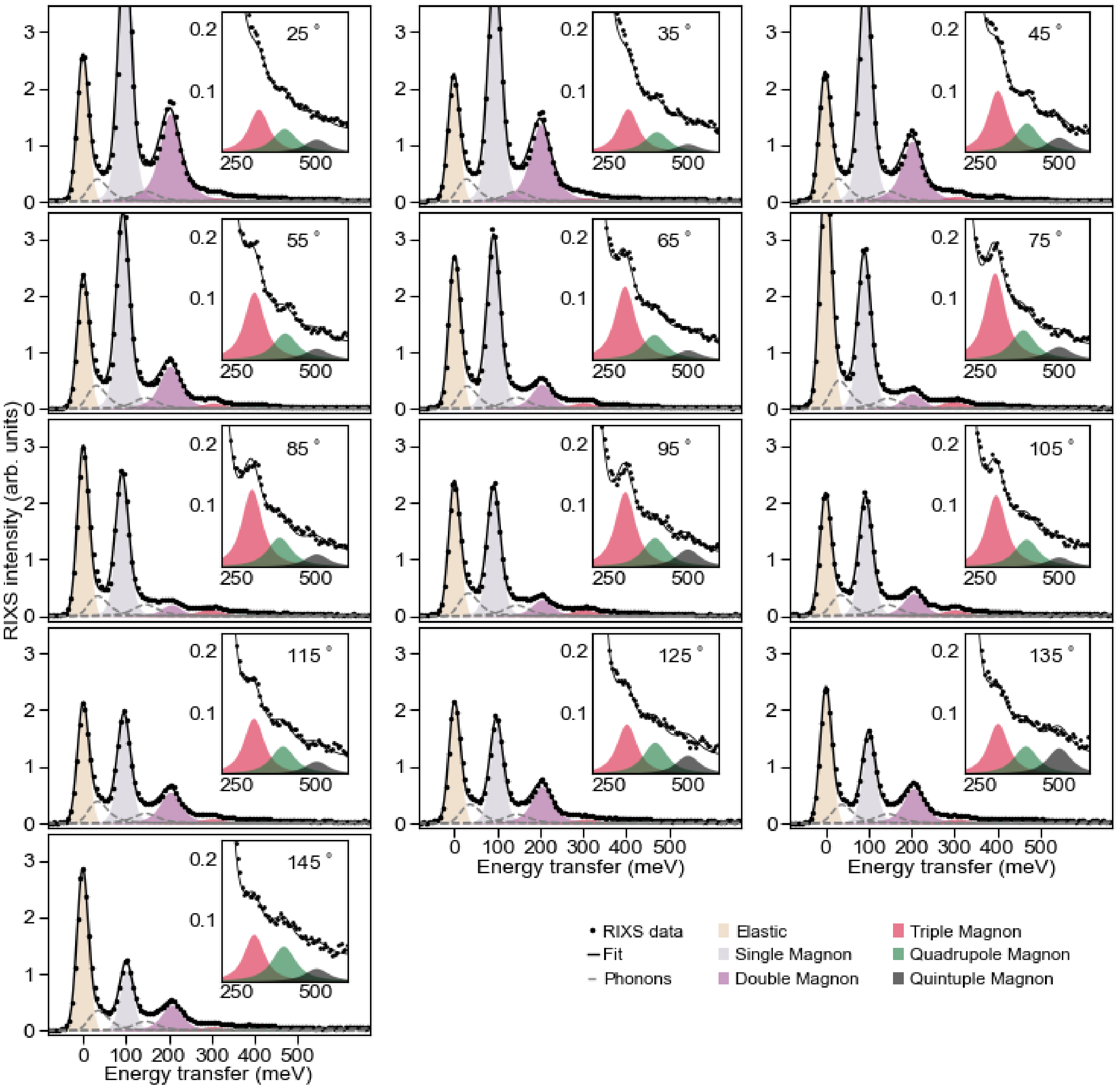

Fig. S1.

Low energy RIXS spectra at E_1 of XAS along with fitted profiles for π polarization and varying α. The insets show the triple- (red), quadrupole- (green) and quintuple- (black) magnon excitations.

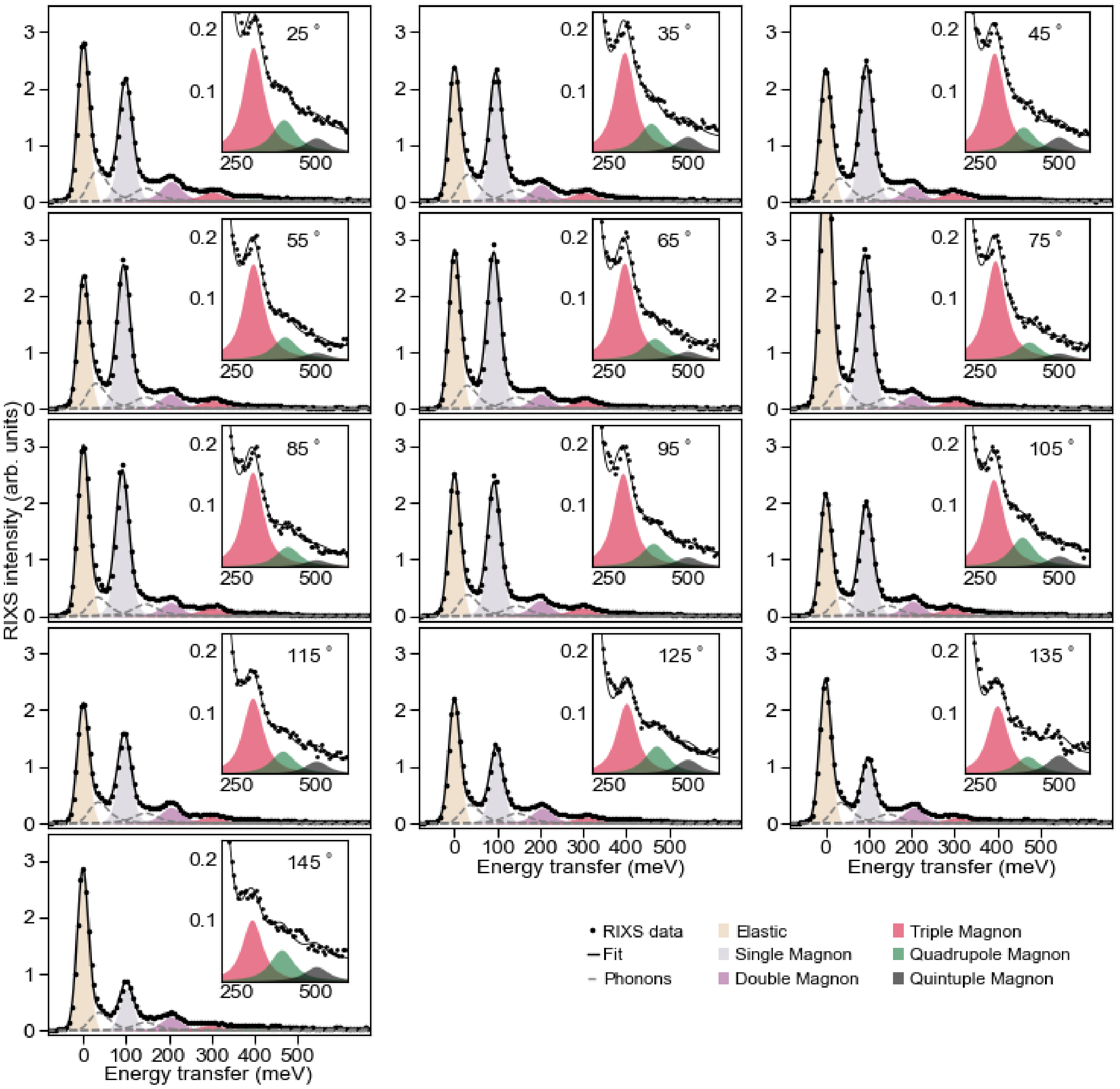

Fig. S2.

Low energy RIXS spectra at E_1 of XAS along with fitted profiles for σ polarization and varying α. The insets show the triple- (red), quadrupole- (green) and quintuple- (black) magnon excitations.

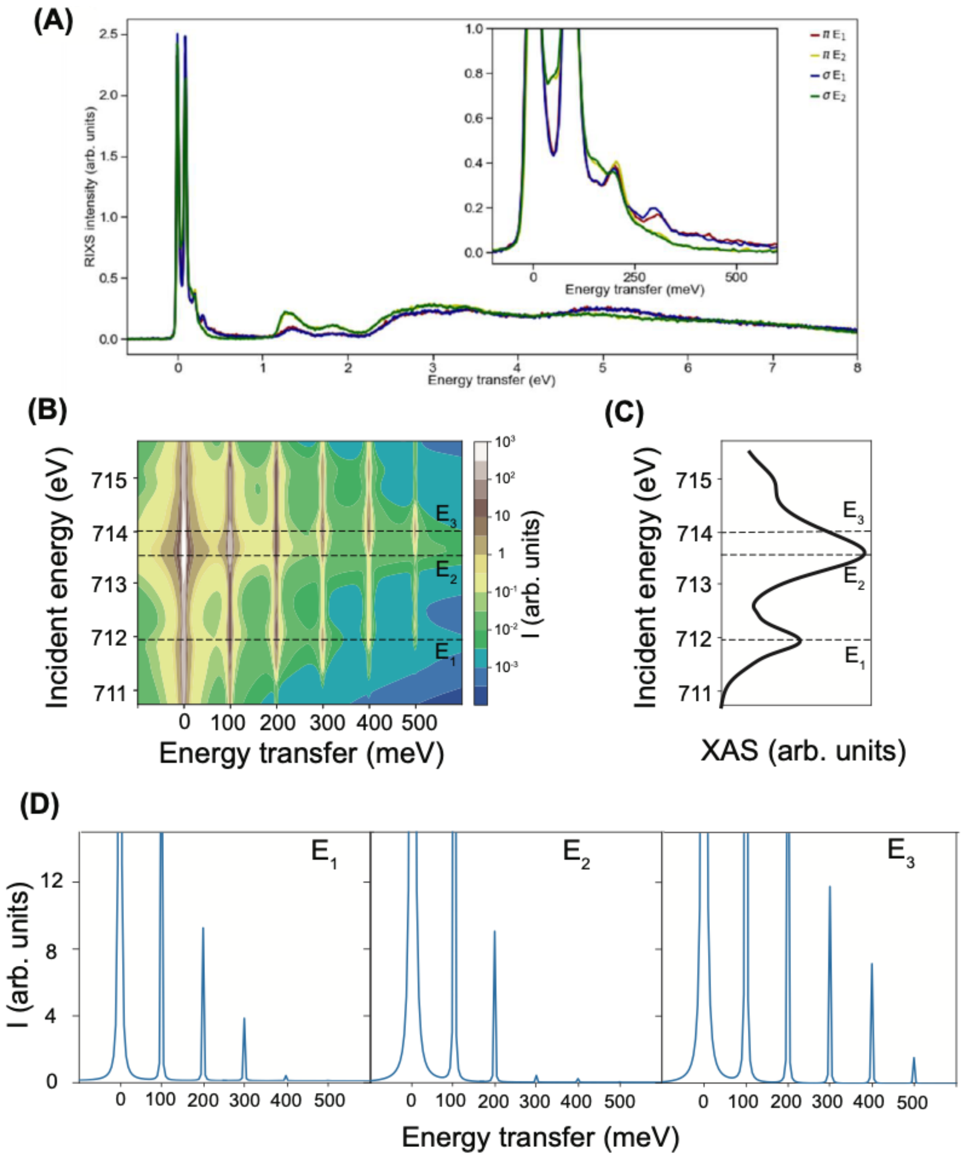

Fig. S3.

(A) Experimental RIXS spectra showing high energy transfer features at E_1 and E_2 of XAS for π and σ polarization at α=95°. Inset shows the low energy transfer features. Features close to 43 meV and 150 meV are clearly visible for incident energy of E_2. However, the multi-magnons are not observed because the incident energy that maximizes the absorption is not the same energy that maximizes the intensity of the multi-magnon. This can be seen in the subsequent panels. (B) Calculated Fe^{3+} RIXS map in Fe_2O_3. The dashed lines show the three energy cuts E_1, E_2 and E_3 which is a virtual cut that maximized the intensity of the multi-magnons. (C) Calculated XAS showing the energy positions of the three energy cuts with respect to the two XAS L_3 peaks. (D) RIXS cuts at the three incident energies E_1, E_2 and E_3 where it can be seen that to observe the multi-magnons the incident energy has to be detuned from the 2nd absorption peak (E_2) to E_3.

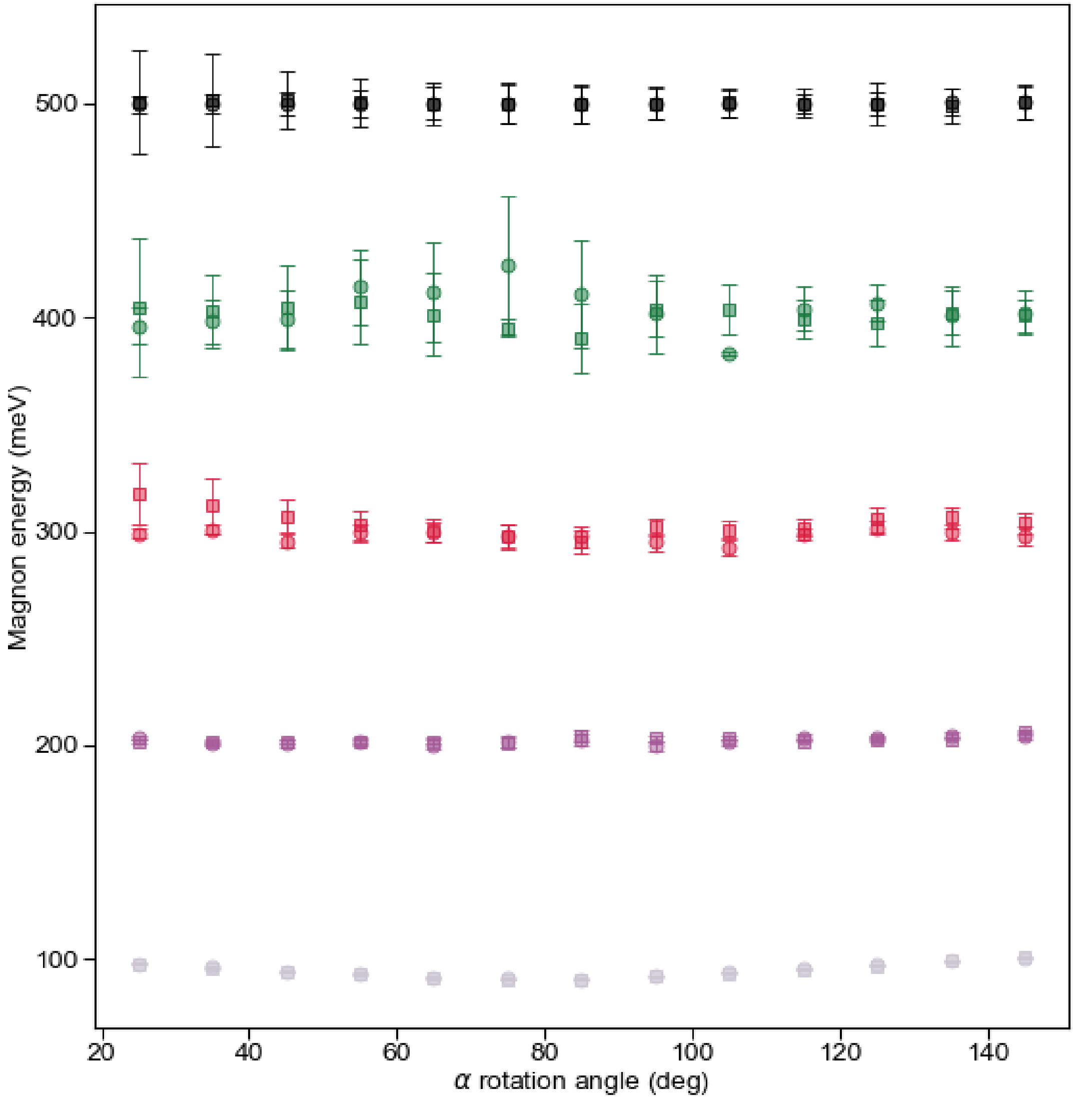

Fig. S4.

The energies of single-, double-, triple-, quadrupole- and quintuple-magnon excitations extracted from fitting of RIXS data at E_1 of RIXS data for π (squares) and σ (circles) polarisation as described in Methods. The error bars of quintuple-magnon excitations shown are least square fitted energy position errors of the triple magnon)*(5/3).

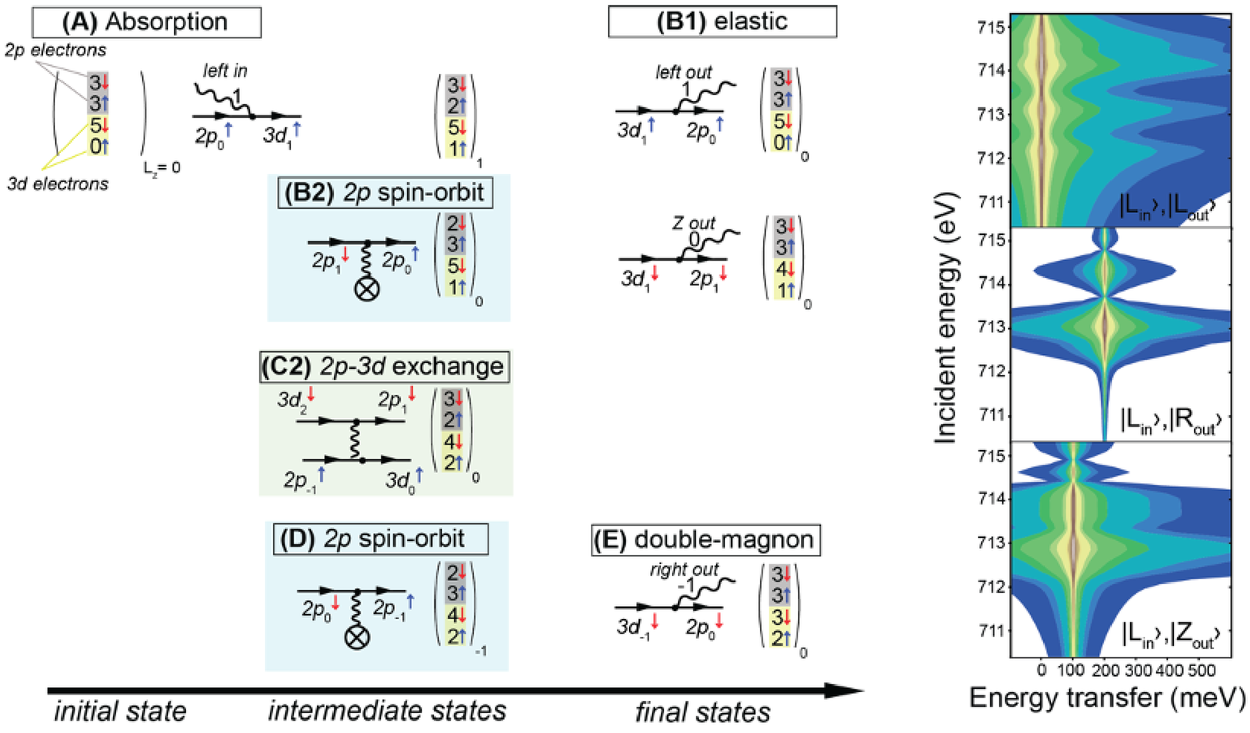

Fig. S5.

Left: Schematic representation of the mechanism of single- and double-magnons by *2p3d* RIXS in the absence of crystal-field. The initial state vector is shown in the upper left corner comprising of the *2p* (gray) and *3d* (yellow) orbitals participating in the RIXS process. The spin of the electrons is depicted by the colored arrows (red = down, blue = up). We follow the fate of a *2p→3d* excitation created by the absorption of a left polarized photon (A) through a cascade of 2p spin-orbit coupling and *2p-3d* exchange interaction through the steps from (B) to (E). It can be concluded that only double-magnons can be excited in the absence of crystal-field.

Right: Fe^{3+} *2p3d* calculations performed without taking into consideration crystal field for circular left polarized incoming beam. The outgoing beam polarization is: circular left, circular right, and Z-polarized from top to bottom respectively.

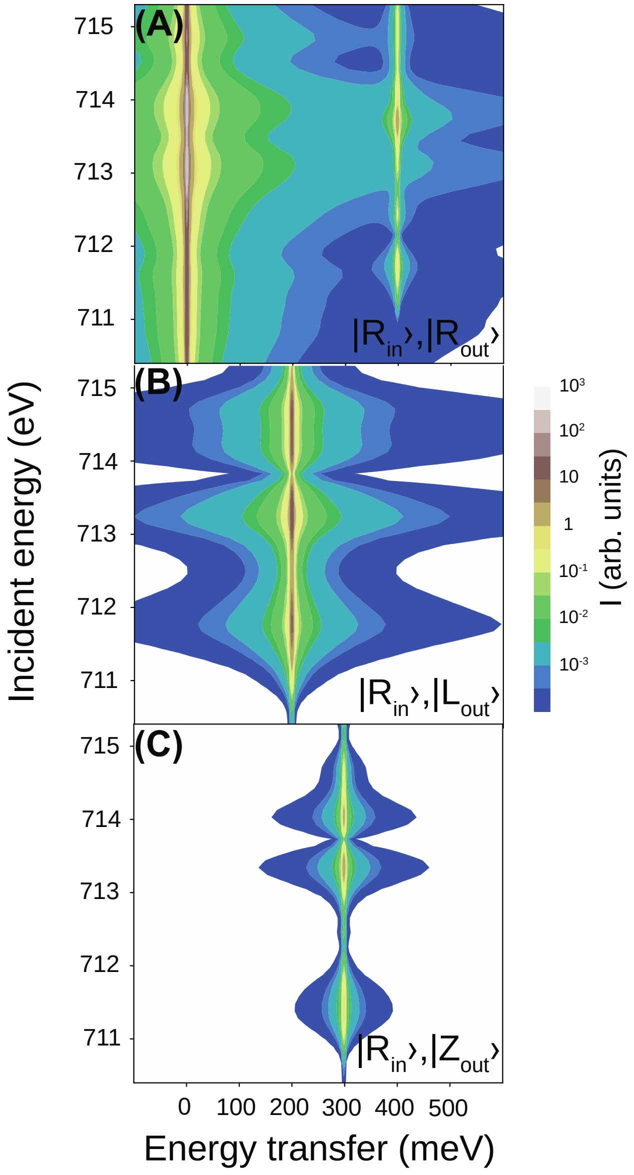

Fig. S6.

Fe^{3+} *2p3d* RIXS calculations performed for circular right polarized incoming beam. The outgoing beam polarization is: (A) circular right, (B) circular left, and (C) Z-polarized.

Parameter	Initials state (eV)	Final state (eV)
$F_{dd}^{(2)}$	8.43	8.97
$F_{dd}^{(4)}$	5.27	5.62
$F_{pd}^{(2)}$		5.96
$G_{pd}^{(1)}$		4.45
$G_{pd}^{(3)}$		2.53
SOC_d	0.059	0.074
SOC_p		8.2
10Dq	1.5	1.5
Jexch	0.1	0.1

Table S1.

Parameters used for the *2p3d* RIXS calculation of Fe^{3+} in α-Fe_2O_3.
